# The Recognition of Scientific Research

**Published:** 1893-02-04

**Authors:** 


### Contemporary Theory and Research.

### THE RECOGNITION OF SCIENTIFIC RESEARCH.

The award of the annual prizes last month by the French Académie des Sciences provokes the inquiry why no similar stimulus to scientific research, not directly utilitarian, exists in England. There is, of course, no lack of prizes for the young student, but when once a man has done with the schools, public acknowledgment of his services to the study of medicine is rare indeed. A man devotes years to the working out of some anatomical or physiological problem, sacrificing for its sake every short cut to fame or fortune. Then with some difficulty, probably at some considerable expense, he finds a publisher to bring his work before the world, and the result is that by all but the dozen men who are working something on the same lines the work is entirely ignored, and the writer remains in his original obscurity. A dozen lines of comment in a medical journal is the sum of his recognition by the medical profession.

"They order these things better in France," where research is honoured even in its tentatory stages, and its progress annually recognised by competent authority; such recognition, it cannot be too strongly urged, is the really valuable point in the award of these prizes, of which we furnish some brief account.

The Prix Montyon for medical and surgical researches was awarded in all to eight persons for work laid before the committee.

1. Dr. Farabeuf, the anatomist, and Dr. Varnier, the celebrated accoucheur, have produced in collaboration a standard work entitled, "Introduction to the Clinical Study and Practice of Midwifery." Though the French obstetrical school is justly renowned, this work is the first in which obsteteric anatomy has been exhaustively treated.

2. M. Emile Javal has distinguished himself by a memoir on the ophthalmometer, an instrument of his own invention. This is not only a clinical instrument more rapid and more exact in operation than others of the same kind, but a physiological instrument which has largely developed the knowledge of optics. M. Javal has devoted his life to researches of this description. By means of the ophthalmometer oculists can determine instantly with the greatest ease the position and seat of astigmatism of the cornea, a knowledge all the more precious from the frequency of the disease.

3. M. Lucas Championnière presents a treatise on "The Radical Cure of Hernia." An adept in antiseptic surgery, and one of the principal agents for its popularisation in France, M. Championnière here confines himself to a record of his personal experience in the radical cure of this complaint. Admitting that in certain cases his treatment is impracticable, he furnishes a record of 275 cases in which it was successful.

4. Drs. Kelsh and Antony owe their award to an able report on "Influenza (La Grippe) in the French Army." The points touched upon are the rapid spread of the epidemic; the numerous points from which the infection simultaneously arose; its later diffusion by contagion; its propagation under the most varied atmospheric conditions; the extraordinary mortality among the troops in Algeria; and the pyæmic and rheumatic complications to which the diseases gave rise.

5. M. Redard presents a standard work illustrated by nearly 800 plates on "Orthopædic Surgery," remarkable as being both complete and concise.

6. M. Pitres presents "Clinical Lectures on Hysteria and Hypnotism," based on direct personal observation.

The Prix Pourat is awarded yearly for physiological research. The subject for 1892 was "Experimental and Clinical Researches into the Inhibitory Phenomena of Nervous Shock," and the prize was awarded to M. Roger. "Nervous shock has been the object of very numerous works," says the report, "especially in England, but the part played by inhibition has been scarcely studied except with regard to cardiac syncope. The most important point consists in a special influence of the nervous system, determining the inhibition of interchange between the tissues and the blood, to which may be ascribed the lowering of the temperament, the red colour of the blood in the veins, and numerous other phenomena." This inhibition is proved by the fact that frogs in a state of shock experience no toxic effects from the injection of strychnine into the blood.

It will be seen from this summary of the awards that the works presented were of the most varied character, and the recipients of the prizes men widely differing in standing and attainments. Such a formal gathering up as it were of the scientific fruits of the year, as attempted by the French Academy of Science, can hardly fail to exercise a most salutary effect upon the profession at large, not only in the encouragement of original research, but in the advancement of the general knowledge of particular achievement.

